# Central line‐associated *Rhodotorula mucilaginosa* fungemia in an immunocompetent host: Case report and review of the literature

**DOI:** 10.1002/ccr3.3969

**Published:** 2021-02-20

**Authors:** Wael Goravey, Gawahir A. Ali, Fatma Abid, Emad B. Ibrahim, Muna A. Al Maslamani, Hamad Abdel Hadi

**Affiliations:** ^1^ Department of Infectious Diseases Communicable Diseases Centre Hamad Medical Corporation Doha Qatar; ^2^ Microbiology Lab Department of DLMP HMC Doha Qatar

**Keywords:** Amphotericin, central venous catheter, fungemia, *Rhodotorula mucilaginosa*

## Abstract

*Rhodotorula mucilaginosa* is an emerging fungal infection with the ability of biofilms formation. The identification of *R mucilaginosa* fungemia should trigger reflexes of prompt central venous line removal and using Amphotericin therapy.

## Graphical abstract

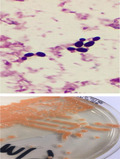

## 1 INTRODUCTION

The fungus *Rhodotorula mucilaginosa* is emerging as a pathogen with high resistance profile and considerable morbidity and mortality, particularly in immunocompromised hosts. We describe a case of CVC‐related *R mucilaginosa* fungemia in an immunocompetent host and appropriate early antifungal selection to prevent potential consequences.

Rhodotorula species are commensal fungi belonging to the family of Sporidiobolaceae with *R mucilaginosa*, *R* *glutinis*, and *R* *minuta* are the most clinical encountered species typically, causing invasive infections in patients with hematological malignancies and others immune dysfunction.^1^, ^2^ In addition to immune‐compromised hosts, the presence of central venous catheters (CVC) is the most important contributing factor despite some patients have reported no such identifiable risk.^3^ The clinical assessment and timely identification of *R mucilaginosa* infection are of paramount importance with the need to remove the CVC and selecting appropriate antifungal treatment, preferably the broad Amphotericin therapy to avoid treatment failure.^4^

We describe a case of CVC‐related *R mucilaginosa* fungemia in an immunocompetent host with immediate removal of CVC and appropriate early antifungal selection to prevented described potential morbidity and mortality associated with *R mucilaginosa* fungemia. Besides, we reviewed the literature for *R mucilaginosa* fungemia in immunocompetent adult patients.

## 2 CASE DESCRIPTION

**FIGURE 1:**
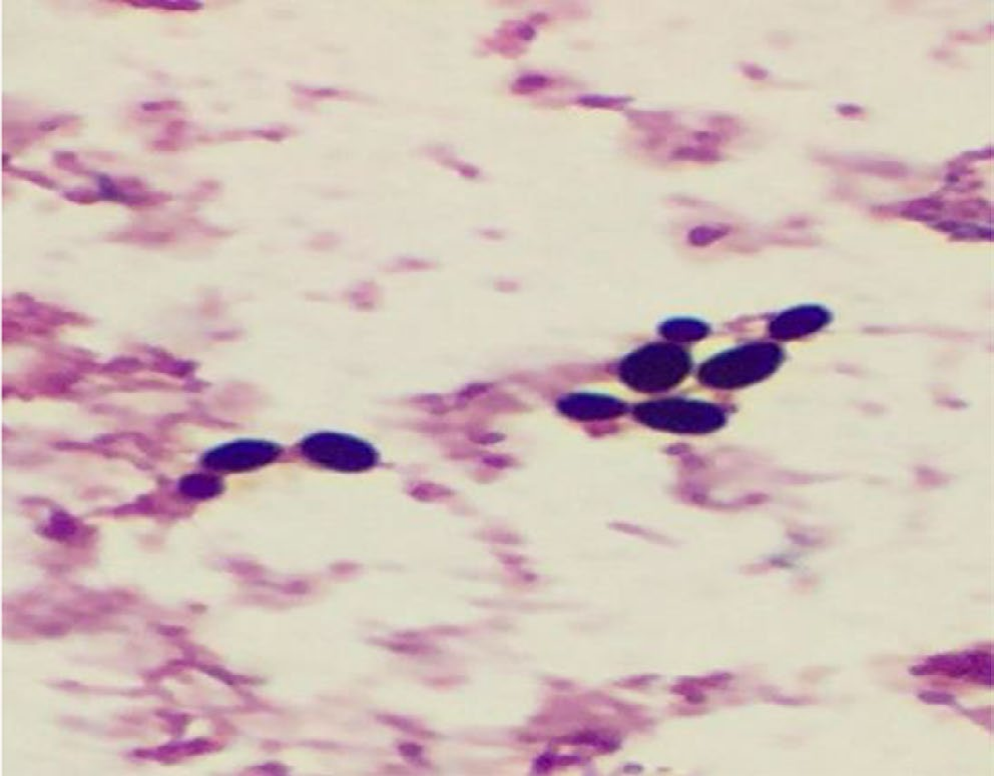
Gram stain revealed oval budding yeast

**FIGURE 2:**
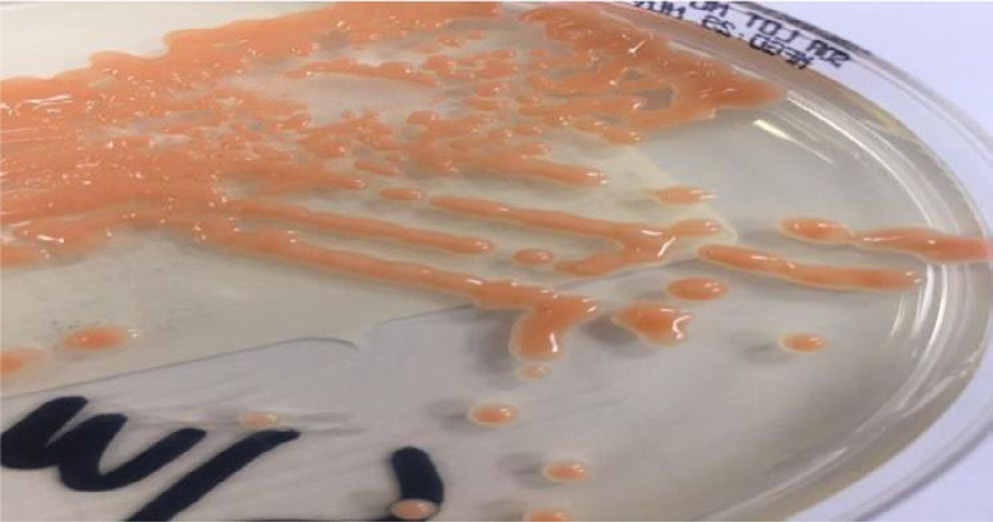
Salmon pink colonies in Sabouraud medium

An 85‐year‐old male with severe Alzheimer's disease and complete dependency on everyday activities. He presented to our emergency department with fever, shortness of breath, and left‐sided chest infiltrates. He was managed as hospital‐acquired pneumonia since he was recently discharged from another healthcare facility. Sputum cultures grew multidrug resistant Pseudomonas aeruginosa (MDRPSA) sensitive to Tazocin, Gentamycin, and Meropenem only. Five days following completing his antibiotics course, and stabilization of his clinical condition, the patient suddenly developed severe septic shock necessitating admission to the intensive care unit (ICU) with new a right‐sided middle zone infiltrate. The patient was treated for severe pneumonia, and antibiotics were upgraded to Meropenum, intubated, vasopressor initiated, monitored, and supported through the internal right jugular central line. His hemodynamic status and inflammatory markers quickly improved; however, fiver days into this ICU admission, he developed a new fever of 38.30°C without obvious source and clean lines with the complete blood counts showed normal hemoglobin, white blood cells of 16.6 × 109/L (4.‐11.0), neutrophils of 12.5 × 109/L (1.5‐8.0), and platelet count of 220 × 109/L (150‐450). The blood cultures from the peripheral and central lines were taken, and gram stains revealed oval budding yeast from one bottle of the central lines following 72 hours incubation, Figure 1. The right internal jugular line was removed, and Anidulafungin was immediately started empirically to cover presumed Candida infection. The patient continued to spike a fever, and the following day, the mass spectrometry assisted by flight time desorption/ionization matrix (MALDI TOF‐MS) identified the yeast as *R mucilaginosa* and later confirmed upon culture with its characteristically smooth Salmon pink colonies in Sabouraud medium, Figure 2. Following correct identification of the rare fungus and search of its antifungal sensitivities, treatment was switched to liposomal Amphotericin B 5 mg/kg/d with rapid defervescence of the fever within 24 hours to complete a 14‐day course. The success of the intervention has been later confirmed, when the isolated *R mucilaginosa* proofed to be sensitive to Amphotericin and resistant to all other agents. To complete the management, repeat cultures were sterile and an echocardiogram showed no valvular vegetations, and the patient stepped down uneventfully from the ICU.

## 3 DISCUSSION

Rhodotorula species are commensal organisms that are commonly encountered from human nonsterile sites like skin, sputum, urine, vagina, and gastrointestinal tracts.^5^, ^6^ The pathogen is an opportunistic infection capable of possessing invasive characteristics in the presence of immunocompromised hosts, particularly in the presence of solid neoplasms, immune suppression, or hematological malignancies.^7^

Rhodotorula fungemia was reported in 48.6% of all Rhodotorula‐related infection in a recent review with *R mucilaginosa* is the most causative species, while the presence of CVC was encountered in 81.6% of the cases.^8^ The majority of *R mucilaginosa* cases in the literature are from patients with immune dysfunction while only a few cases are immunocompetent.^9^, ^10^, ^11^, ^12^, ^13^, ^14^

Our patient is an elderly without an obvious immunosuppression status, but there are multiple risk factors which put him for acquiring invasive fungal infections, hospital‐acquired pneumonia with a multidrug‐resistant organism, treatment with abroad spectrum antimicrobial therapy, a recent admission to critical care unit with access through CVC, urinary catheterization all significant contributory factors to invasive fungal infections.^2^ The fact that *R mucilaginosa* was grown from the CVC and not peripherally with a plausible time frame supports that the CVS was the port of entry as commonly described in the literature and likely the contributory factor to the development of the fungemia. In addition to that Rhodotorula spp has high avidity of forming biofilms around medical devices, leading to significant mortalities and devise loss.^15^ The development of a new fever while our patient covered with potent broad‐spectrum antibiotics was the hint of fungal infection in this patient. The clinical judgment of covering with Anidulafungin was appropriate to cover for candidemia in the ICU setting; however, nonresponse should raise suspicion on an alternative pathology. The differentiation between Candida and Rhodotorula spp is based upon gram stain is very challenging, and rarely possible as both show oval budding yeast with rudimentary pseudohyphae.^16^ Some laboratories use the positive urease test as a quick pointer to differentiate between the Rhodotorula spp and Candida app, but it should be noted the limitation of such approaches, including the positive yield of the test with some Candida spp.^17^

The identification of *R mucilaginosa* fungemia is a crucial step in management and should be followed by actively looking and removing any medical devices, in addition, to change the antifungal cover to Amphotericin.^4^ The idea that without removing the offending device the organism will continue to grow into the bloodstream leading to significant morbidities and mortalities. Of note, some patients have been managed by the removal of CVC only without antifungal administration because of the low pathogen virulence.^8^ Despite the immediate removal of the CVC when yeast was identified in the blood, our patient continued to fever which only settled after switching to Amphotericin. Amphotericin yields the lowest MIC to Rhodotorula while an intrinsic mechanism of resistance to Fluconazole was suggested based on it is high MIC. Echinocandin is not a choice for the treatment as it has poor activity while Posaconazole or Voriconazole remains an option without much available data.^18^, ^19^

The mortality of Rhodotorula spp fungemia has been reported around 10% with the complication of sepsis, shock, and organs dysfunction.^8^

Our patient finished a 2‐week course of Amphotericin, which is the only available option of treatment based on the sensitivity of the isolate. The patient continued to improve without complications and repeated blood cultures were sterile.

Our search of the literature yielded a total of 7 cases of *R mucilaginosa* fungemia in immunocompetent adult patients (Table 1).^9^, ^10^, ^11^, ^12^, ^13^, ^14^ The majority of cases were males, and the median age was 50 years. The notable risk factors for most of the cases were the ICU admission and use of broad‐spectrum antibiotics. Of the seven cases reviewed, *R mucilaginosa* identification using sequencing of internal transcribed spacer ITS1/ITS2 regions of rDNA was used in only one case.^12^ Amphotericin B alone was used in two cases, while Fluconazole was added to Amphotericin B in another two cases.^9^, ^11^, ^12^, ^13^ Of note, Fluconazole alone was used in two cases.^9^, ^10^ The strategy of CVC removal was applied in six out of the 7 cases. More than half of the patients survived, though the outcome was not always available. Important to note that, the death in the 88‐year‐old man was before the identification of the *R mucilaginosa* and receiving an effective antifungal therapy.^14^

**TABLE 1:** Summary of previously reported adult cases of *R mucilaginosa* fungemia in an immunocompetent host

Study	Gender/Age, y	ICU admission	Broad‐spectrum antibiotics	Method of identification	Antifungal therapy	Removal of CVC	Outcome
Zaas et al^9^	No data available/32	No data available	No data available	API 20C AUX system	Fluconazole	Yes	No data available
Zaas et al^9^	No data available/35	No data available	No data available	API 20C AUX system	Amphotericin B+ Fluconazole	Yes	No data available
Duggal et al^11^	F/50	Yes	Yes	API 20C system	Amphotericin B	Yes	Alive
Villar et al^10^	F/62	Yes	Yes	API 20C AUX system	Fluconazole	Yes	Alive
Kim et al^12^	M/77	Yes	Yes	API 20C AUX system+ rDNA D1/D2 domain sequence analysis	Amphotericin B+ Fluconazole	Yes	Alive
Pereira et al^13^	M/39	No	No	No data available	Amphotericin B	‐	Alive
Falces‐Romero et al^14^	M/88	Yes	Yes	MALDI TOF‐MS	Fluconazole, died before the identification	Yes	Death
Our case, 2020	M/85	Yes	Yes	MALDI TOF‐MS, Billerica, MA, USA	Amphotericin B	Yes	Alive

## 4 CONCLUSION

*Rhodotorula mucilaginosa* fungemia is an emerging invasive pathogen that required a high index of suspicion, particularly in critical care units with central lines, and multiple risk factors. The identification of *R mucilaginosa* fungemia should trigger immediate steps of prompt removal of the central lines, using Amphotericin B as the first option of therapy and addressing the reversal of risk factors.

## CONFLICT OF INTEREST

None declared.

## AUTHOR CONTRIBUTION

WG: contributed to data acquisition and manuscript preparation; GA: contributed to data acquisition and manuscript writing; FA and EB: contributed to data acquisition and microbiology reports; HA and MA: supervised all the aspects and contributed to final manuscript editing.

## ETHICAL APPROVAL

Ethics approval and patients' consent was obtained for the publication of this case reports and all accompanying images. Permission was obtained to publish the case reports from the institutional review board which is in line with international standards.

## Data Availability

The authors confirm that the datasets supporting the findings of this case are available from the corresponding author upon request.
